# Spatio-Temporal distribution characteristics and driving factors of traditional villages in the Yellow River Basin

**DOI:** 10.1371/journal.pone.0303396

**Published:** 2024-05-21

**Authors:** Yuehao Huang, Qianming Xue

**Affiliations:** School of Architecture and Urban Planning, Lanzhou Jiaotong University, Lanzhou, China; Universita degli Studi del Molise, ITALY

## Abstract

Currently, research on traditional villages mainly focuses on the current development status and evolutionary trends in specific regions, with relatively limited studies from a macroscopic and holistic perspective on the spatiotemporal evolution of traditional villages. Therefore, this study selects traditional villages in the Yellow River Basin (YRB) as the research object. By analyzing the spatiotemporal distribution characteristics and driving factors of traditional villages (TVs) in the basin, it aims to further promote high-quality development in the YRB and protect traditional cultural resources. Based on data from 892 village points of the first to sixth batches of TVs in the YRB, ArcGIS 10.8 spatial analysis techniques were employed to analyze the overall spatial pattern of TVs in the YRB. The results indicate: (1) In the basin, TVs are more numerous in the east than the west and more in the south than the north, forming clusters and contiguous distributions, with dense areas primarily in the upstream regions dominated by Qinghai Province and the midstream areas along the Shanxi-Shaanxi coast. (2) The number and scale of TVs in the basin generally exhibit an increasing trend, with imbalanced provincial distribution. More recent years show a more balanced distribution of villages and proportions, with a higher number of villages in the mountainous and plateau regions of the basin. (3) The layout center of TVs within the basin evolves with each batch, showing a migration pattern from north to south, back to north, and finally east to west. (4) The interaction of natural and social factors plays a synergistic role in driving the spatiotemporal distribution pattern of TVs. Among these, natural geographical factors are the primary factors. TVs are more commonly found in regions with low altitude sunny slopes, mild climate, abundant precipitation, proximity to ancient roads and rivers, gentle slopes, and soil predominantly comprising loess, brown earth, and alluvial soils. The cultural environment is a secondary factor, with TVs often located in areas with larger populations, developed economies, and rich cultural heritage.

## 1. Introduction

TVs, also known as "ancient villages", refer to villages and settlements with a relatively long history, developed structures, and relatively complete preservation [1]. This year marks the 12th year of China’s Traditional village preservation initiative. As of February 2024, China has announced six batches of national traditional village protection lists, with 8155 villages now included in China’s Traditional village Catalog, forming the world’s largest and most complete protection group of agrarian civilization heritage [2]. China, having the highest number of TVs globally, sees these villages as a unique cultural landscape, rich and diverse in resources, and broadly distributed in space, recording the temporal process of human-nature interaction and bearing the local rural culture and historical memory [3, 4]. However, with the rapid advancement and intense impact of urbanization, industrialization, and modernization worldwide, rural decline is increasingly becoming a global issue [5]. The inactivity and decline of TVs have gradually garnered attention from governments and societies. In recent years, the continuous transformation of traditional agricultural production methods, the homogenization of agricultural culture and customs, and the gradual disappearance of traditional residential buildings and architectural features pose threats to the revitalization and sustainable development of TVs. Furthermore, the massive rural population migration to cities exacerbates the hollowing and aging of villages, leading to issues like "abandoned homes and buildings", "neglected structures", and "idle facilities" in traditional village preservation and development. As a country with a thousand-year history of agrarian civilization, TVs not only encapsulate historical memory but also reflect the progress of Chinese civilization. They also promote regional high-quality development and the preservation of traditional cultural resources. Therefore, alongside urbanization and industrialization, villages as traditional settlements and preservations have gradually become a new focal point in research, promoting regional sustainable development and urban-rural integrated development. Given this, as TVs still face numerous difficulties and challenges and lack spatial characteristics and their evolutionary processes in regional sustainable and high-quality development, it is necessary to study the spatial characteristics of TVs from a more macroscopic and holistic regional perspective. Analyzing the development direction, evolving trends, and influencing factors of TVs in the region is also the research purpose and focus of this paper.

In recent years, academic research has focused significantly on the protection of traditional and ancient settlements. The main areas of study include the protection of ancient settlement sites, excavation and preservation of early settlements, conservation and restoration of architectural heritage, rural protection and utilization, and preservation of settlement cultural landscapes. Examples include geological archaeological studies of contrasting sites like Chalcolithic Borduşani-Popină, Borcea River, Romania, and Viking Age Heimdaljordet, Vestfold, Norway [6], excavations at’ Aoa Valley, Tutuila Island, American Samoa [7], preservation of traditional Korean settlement architecture [8], century-long development and protection of Shirakawa village Ogimachi in Japan and Kryvorivnia village in Ukraine [9], and the cultural landscape of Minangkabau traditional settlements in Nagari Sijunjung [10]. These studies summarize historical experiences in settlement protection and combine them with rural development and protection, and preservation of native architecture and landscapes, aiming to find new feasible approaches for the preservation and development of traditional settlements. Regarding content, research on TVs mainly focuses on spatial distribution characteristics and their influencing factors [11–16], attempting to reveal the spatial aggregation characteristics and driving factors of TVs through a series of spatial analyses [17–21], thereby promoting village protection and integrated urban-rural development. In order to further promote the sustainable development of TVs, some scholars have conducted appropriate technical evaluations of them, studying the spatial distribution of cultural heritage through digital technology, aiming to comprehensively support the revitalization of ancient villages [22, 23]. Additionally, some scholars use the distribution of rural settlements within the region as a breakthrough point, reflecting a certain spatial pattern through these settlements, demonstrating the coordination of rural settlement distribution with various environmental and human factors, in order to maximize profits from agricultural land [24]. In terms of methodology, analyses of traditional village distribution patterns are primarily conducted using multi-scale geographically weighted regression [25], GWR, and geographical detectors [26]. Additionally, scholars have explored TVs in different regions from various perspectives, mainly focusing on protection and development [27–29]. Initially, Chinese academic research on TVs primarily focused on living environments [30–33], cultural landscape genetics [34, 35], and spatial patterns and causes [36–38]. To further promote the preservation and development of TVs, the Ministry of Housing and Urban-Rural Development initiated the investigation of China’s traditional catalog in 2012. In 2017, the government proposed the protection of TVs, traditional residences, and historic cultural towns, providing significant guidance for the preservation and development of TVs; the same year, the introduction of the rural revitalization strategy injected new vitality into the protection and utilization of TVs [39, 40]. The successive introduction of related policies not only promoted the protection and development of TVs but also drove regional high-quality and sustainable development. With the development of rural cultural tourism in China, scholarly research on TVs has witnessed a new wave of attention. As research deepens, recent studies on TVs have focused on both traditional and new perspectives, with the main content summarized as follows: (1) In terms of the spatial distribution of TVs, scholars have expanded the scope of selected areas based on previous studies of spatial patterns and causes, conducting in-depth research on the driving factors and differentiation rules of spatial distribution of TVs in various regions [41–43]. (2) Regarding the protection and development of TVs, scholars have placed more emphasis on the protection of village style [44], architectural cultural heritage [45, 46], and concentrated contiguous protection [47, 48]. Addressing how TVs should be protected and utilized, scholars have linked TVs with intangible cultural heritage [49, 50], landscapes [51, 52], and some have researched the vitality [53], local legislation [54], and adaptability [55] of traditional village protection and development. (3) Additionally, perspectives on traditional village research have diversified. Recently, scholars have studied TVs from angles such as rural revitalization [55], "human-industry-location" elements [56], three livelihoods [57], anthropology [58], and regionalism [59]. Theoretical and methodological innovations continue, with applications like AHP-Entropy Method [60], H-I-F Element Synergy [61], SolVES [62] being introduced in the field of traditional village research, and new research directions like genetic maps [63, 64], landscape patterns [65] are being explored. Overall, Chinese scholars’ research on TVs has mainly concentrated on the current development and evolving trends within specific regions or locales, while macroscopic and holistic studies on the temporal and spatial evolution of TVs remain superficial.

In summary, existing policies and literature highlight some pressing issues in the study of TVs. A comprehensive analysis of the spatial characteristics of TVs within a region and an in-depth exploration of the various factors affecting their spatial distribution are fundamental and key to achieving the protection, inheritance, and sustainable development of these villages. The Yellow River, the cradle of Chinese civilization and a hub of excellent traditional and ethnic cultures, is home to numerous well-preserved TVs. Today, the global emphasis on ecological watershed protection and the management of ecological environments has made the study of TVs and settlement cultures significantly relevant to advancing global ecological governance and cultural resource protection. This paper selects six batches of TVs announced by China’s Ministry of Housing and Urban-Rural Development between 2012 and 2024 as the subject of study. It analyzes the temporal and spatial evolution characteristics of each phase and the overall driving factors affecting spatial distribution, employing methods such as the nearest neighbor index, kernel density analysis, hot and cold spot analysis, buffer zone analysis, and geographical detectors. This approach aims to align with the national rural revitalization strategy of the new era, providing insights and references for the protection of TVs in the watershed and regional high-quality development.

## 2. Overview of the geographic region

**Fig 1:**
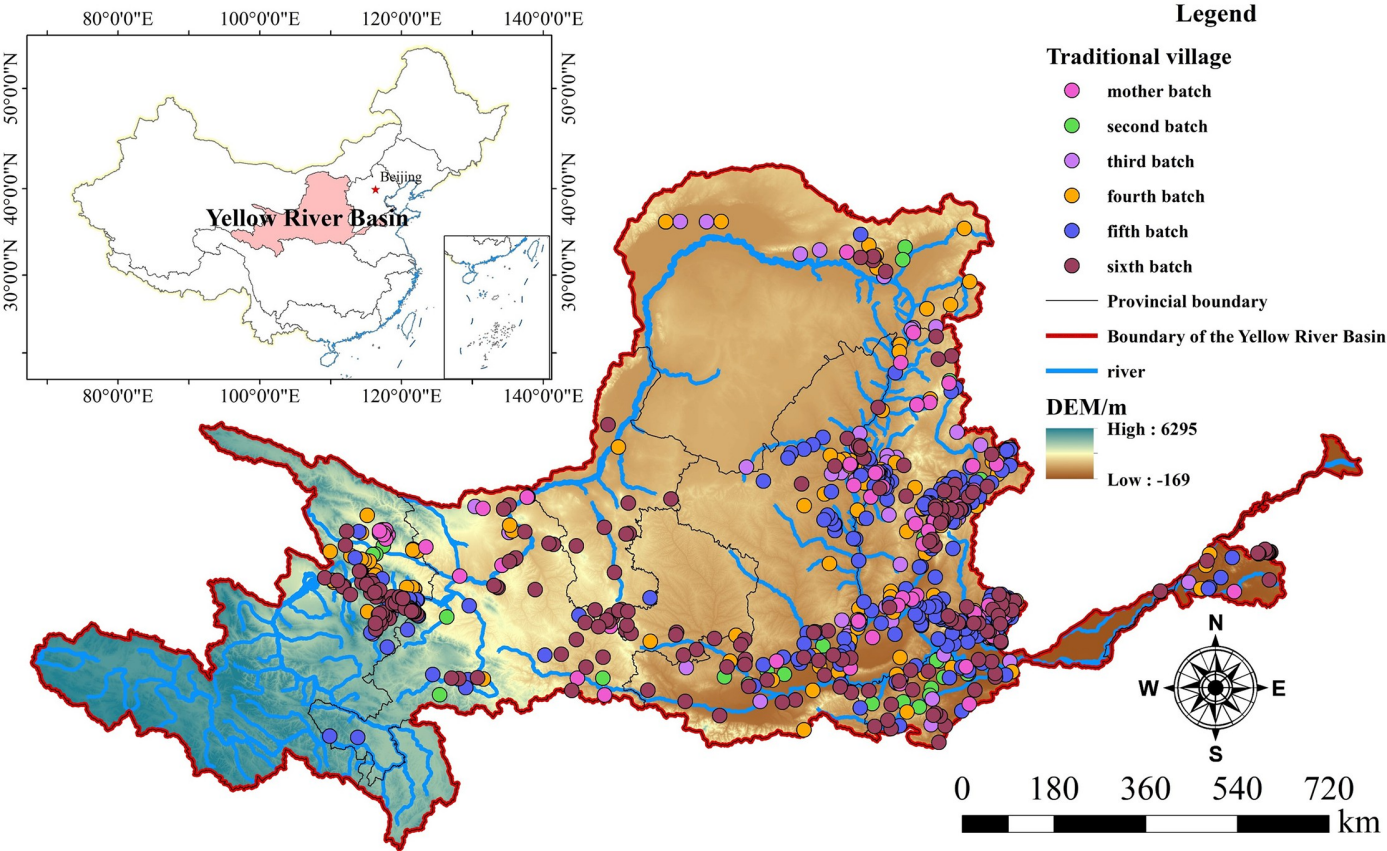
Study area. The maps were generated by ArcGIS 10.8 and were for illustrative purposes only.

The Yellow River, China’s second-longest river and known as the "Mother River", originates from the Bayan Har Mountains in the central-southern part of Qinghai Province. It spans central, eastern, and western regions, covering a large area and flowing through nine provinces and regions including Qinghai, Sichuan, Gansu, Ningxia, Inner Mongolia, Shaanxi, Shanxi, Henan, and Shandong [66]. The YRB studied in this paper is located between 32°10′–41°50′ N and 95°53′–119°05′ E, with an area of 7.95×10^5^ km^2^ [67, 68], accounting for 8% of China’s land area (Fig 1). Most of the basin lies in arid and semi-arid areas, with a predominantly dry climate. Annual precipitation is about 200-600mm, only 44.1% of that in the Yangtze River Basin. Temperatures in the basin decrease from east to west and from south to north, ranging from -4 to 14°C annually, with a high evaporation rate, averaging 1100mm per year. The YRB is the cradle of Chinese civilization, with distinctive regional cultures from upstream to downstream, and is the most concentrated area of TVs in northern China. According to the "Catalog of Traditional Chinese Villages (Batches 1–6)" published by the Ministry of Housing and Urban-Rural Development, the basin encompasses 9 provinces, 83 cities, and 395 counties, with a total of 892 TVs. The concentrated distribution of TVs in the YRB is a true reflection and record of the evolution and transition of settlement civilization in the basin, as well as a historical witness to cultural inheritance and change. The YRB is not only a vital source of water and ecological barrier in northern China but also a hub of cultural heritage, embodying rich ecological cultural concepts and wisdom in human settlement, making it an important carrier for promoting harmonious human-environment interactions and high-quality development in contemporary watersheds.

## 3. Materials and methods

### 3.1 Data sources

The 1–6 batches of TVs mentioned in the text are derived from the national traditional village catalogs published in 2012, 2013, 2014, 2016, 2019, and 2023 by the Ministry of Housing and Urban-Rural Development, Ministry of Culture and Tourism, and Ministry of Finance of China. Currently, there are 892 TVs in the basin, and their geographical coordinates within the YRB were obtained using the Amap Application Programming Interface (API) coordinate system, which were then converted into spatial Polnt of Information (POI). These POI were visualized on the vector map of the YRB using GIS. The vector data of China’s administrative boundaries were based on the GS (2020) 4615 standard map downloaded from the Standard Map Service website of the Ministry of Natural Resources of China (http://bzdt.ch.mnr.gov.cn/index.html), unmodified. Vector data of the spatial extent of the YRB were sourced from the Resource and Environmental Science and Data Center of the Chinese Academy of Sciences (http://www.resdc.cn). Digital Elevation Model (DEM) digital elevation data were obtained from the Geospatial Data Cloud platform of the Chinese Academy of Sciences (https://www.gscloud.cn). Ancient path traffic data were acquired from the Open Street Map (OSP) open-source platform (https://www.openstreetmap.org), and river data from the 1:250,000 national basic geographic database provided by the National Geographic Information Resources Catalogue Service System. Data on temperature (1 km resolution), precipitation (1 km resolution), and elevation (30 m resolution) were obtained from the National Earth System Science Data Center (https://www.geodata.cn). Additionally, the 2022 gross domestic product (GDP) of the nine provinces of Qinghai, Gansu, Ningxia, Sichuan, Inner Mongolia, Shaanxi, Shanxi, Henan, and Shandong were sourced from the China Statistical Yearbook. Data on provincial capitals and prefecture-level cities were obtained from the National Bureau of Statistics of China (http://www.stats.gov.cn), and the seventh population census data from the World Urban Population website (http://www.citypopulation.de).

### 3.2 Research method

#### 3.2.1 Standard Deviation Ellipse

The Standard Deviational Ellipse (SDE) method can be used to clearly observe the distribution characteristics and trends of TVs within the YRB. In the formula, the azimuth angle *θ* represents the spatial distribution direction of the TVs, the long axis of the ellipse indicates the degree of deviation of the TVs from the centroid in the primary direction, and the short axis represents the degree of deviation in the secondary direction. The SDE is mainly used to reveal the changes in the distribution center and the spatial evolutionary trends and directions of the batches 1–6 of TVs in the YRB at different time periods, with the calculation formula as follows:

$$\mathrm{Tan}\theta =\frac{(\sum_{i=1}^{n}{\tilde{x}}_{i}^{2}-\sum_{i=1}^{n}{\tilde{y}}_{i}^{2})+\sqrt{{\left(\sum_{i=1}^{n}{\tilde{x}}_{i}^{2}-\sum_{i=1}^{n}{\tilde{y}}_{i}^{2}\right)}^{2}+4{\left(\sum_{i=1}^{n}{\tilde{x}}_{i}{\tilde{y}}_{i}\right)}^{2}}}{2\sum_{i=1}^{n}{\tilde{x}}_{i}{\tilde{y}}_{i}}$$

$$\sigma_{x}=\sqrt{2}\sqrt{\frac{\sum_{i=1}^{n}{\left({\tilde{\chi }}_{i}\cos\theta -{\tilde{y}}_{i}\sin\theta \right)}^{2}}{n}};\sigma_{y}=\sqrt{2}\sqrt{\frac{{\left(\sum_{i=1}^{n}{\tilde{x}}_{i}\cos\theta -{\tilde{y}}_{i}\sin\theta \right)}^{2}}{n}}$$ (1)

In Eq (1), ${\tilde{x}}_{i}$ and ${\tilde{y}}_{i}$ represent the coordinate deviations from the centroid to the geometric center of the basin, while *σ*_*x*_ and *σ*_*y*_ represent the standard deviations of the ellipse along the *x* and *y* axes, respectively.

#### 3.2.2 Nearest Neighbor Index

The Nearest Neighbor Index (NNI) method is used to study the overall distribution pattern of TVs in the YRB, indicating the degree of proximity between TVs within the basin. The calculation formula is as follows:

$$R=\frac{\overset{¯}{r_{i}}}{\overset{¯}{r_{e}}}=\frac{\overset{¯}{r_{i}}}{2\sqrt{\frac{n}{A}}}$$ (2)

In Eq (2), $\overset{¯}{r_{e}}$ represents the expected mean nearest neighbor distance in a random spatial distribution of TVs; $\overset{¯}{r_{i}}$ represents the actual mean nearest neighbor distance of TVs; *A* is the area of the study region; *n* is the number of TVs distributed within the basin. When *R* = 1, the TVs are randomly distributed within the basin; when *R*<1, the TVs are clustered within the basin; when *R*>1, the TVs are evenly distributed within the basin.

#### 3.2.3 Kernel Density Estimation

The Kernel Density Estimation (KDE) method is primarily used to calculate the distribution density of TVs in the YRB and to identify dense areas of TVs within the basin. The calculation formula is as follows:

$$f(x)=\sum_{i=1}^{n}{\left[1-\frac{{\left(x-x_{i}\right)}^{2}+{\left(y-y_{i}\right)}^{2}}{h^{2}}\right]}^{2}/(\pi nh^{2})$$ (3)

In Eq (3), *x* represents the actual points of TVs; *h* is the search radius within the basin; *n* is the number of traditional village points within the search radius of the basin; each point (*x*_*i*_,*y*_*i*_) continuously diffuses outward from the highest point in space, and the density value becomes zero when the distance from the center reaches the search radius (*h*) to a certain extent. A higher kernel density value *f*(*x*) indicates a greater distribution density of TVs; conversely, a lower kernel density value *f*(*x*) indicates a lower distribution density of TVs.

#### 3.2.4 Buffer analysis

Buffer zone analysis is used to describe the degree of proximity between two objects in the spatial context of the YRB. Focusing on the river system and ancient road transportation of the YRB as the subjects of analysis, and considering TVs as the objects affected by rivers and transportation, this method analyzes the relationship between the river system and the distribution of TVs. The calculation formula for the buffer zone size of a fixed point *A* is as follows:

P={x||d(x,A)≤r} (4)

In Eq (4), *r* is the buffer zone radius of the TVs within the basin; *d* is the Euclidean distance from a planar point *x* to point *A*; *P* is the buffer zone of feature *A*.

#### 3.2.5 Hot spot analysis

Hot spot analysis is a method of analysis and determination within local autocorrelation, primarily used to measure the clustering relationship between each observation unit and its surrounding units. When $G_{i}^{*}>0$, it indicates that the area is a hot spot of positive clustering. When $G_{i}^{*}<0$, it indicates that the area is a hot spot of negative clustering.


$$G_{i}^{*}=\frac{\sum_{j=1}^{n}w_{ij}x_{j}-\bar{x}\sum_{j=1}^{n}w_{ij}}{s^{2}}$$ (5)

In Eq (5), where *i* represents the central feature, *n* is the total number of TVs, *j* represents all features within the neighborhood, *x*_*j*_ is the attribute value of feature *j*, and *w*_*ij*_ is the spatial weight between features *i* and *j*.

#### 3.2.6 Geographical detector

The geographical detector is an analytical tool used for detecting and utilizing spatial heterogeneity. It can identify both the spatial heterogeneity of a single factor and the coupled correlation between two factors. In the text, two detectors from the geographical detector are used: the differentiation detector and the interaction detector, mainly to explore the relationship between TVs and other factors within the YRB. The differentiation detector specifically aims to detect the spatial heterogeneity of TVs and the extent to which factor *x* explains variable *y*, i.e., the degree of spatial differentiation of TVs. The calculation formula is as follows:

$$q=1-\frac{\sum_{h=1}^{L}N_{h}\sigma_{h}^{2}}{N\sigma^{2}}$$ (6)

In Eq (6), The q-value measures the detection power of the independent variable, ranging from [0, 1]. A larger q-value indicates greater explanatory and influential power of factor *x* on the spatial distribution *y* of TVs. Here, *h* = 1, 2, …, L represents the stratification of variable *y* or factor *x*, i.e., categorization or zoning; *N*_*h*_ is the number of units in layer *h*; *N* is the number of units in the basin; $\sigma_{h}^{2}$ is the variance of layer *h*; *σ*^2^ is the variance of *y* values in the basin. The interaction mainly identifies the influence of pairwise interactions between different factors, i.e., detecting whether the interaction of influencing factors *x*_1_ and *x*_2_ enhances, diminishes, or remains independent in their impact and explanatory power on variable *y* [69–71].See Table 1.

**Table 1:** Geo-detector interaction detection type table.

Detection Type	Judgment Standard of Interaction Factor
Nonlinear weakening	*q*(*x*_1_∩*x*_2_)<Min (*q*(*x*_1_),*q*(*x*_2_))
Single-factor nonlinear weakening	Min ($q(x_{1}),q(x_{2}))<q(x_{1}\cap x_{2})$ < Max (*q*(*x*_1_),*q*(*x*_2_))
Two-factor enhancement	*q*(*x*_1_∩*x*_2_)> Max (*q*(*x*_1_),*q*(*x*_2_))
Independence	$q(x_{1}\cap x_{2})=q(x_{1})+q(x_{2})$
Nonlinear enhancement	$q(x_{1}\cap x_{2})>q(x_{1})+q(x_{2})$

## 4. Spatio-Temporal distribution patterns and evolution analysis

### 4.1 Spatio-Temporal distribution characteristics

#### 4.1.1 Spatial distribution characteristics

**Fig 2:**
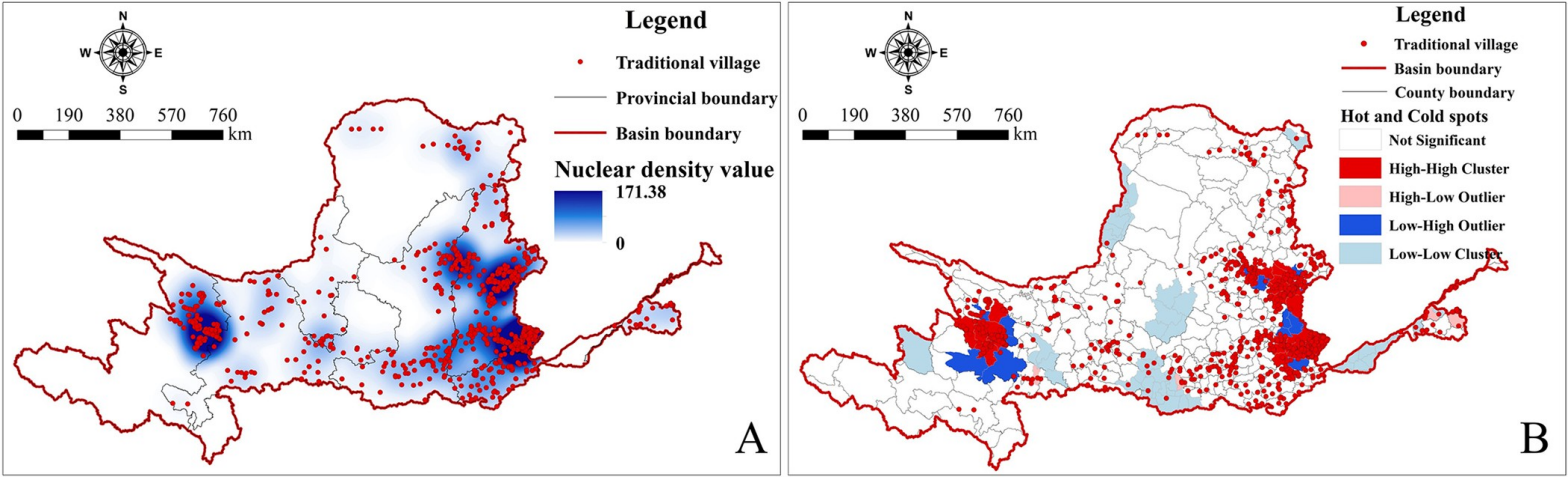
Analysis of spatial distribution density and hot and cold spots. The maps were generated by ArcGIS 10.8 and were for illustrative purposes only.

Using ArcGIS, the average nearest neighbor calculation was performed for TVs within the YRB. According to Formula (1), the average observed distance of TVs is 7.530km, with an expected average distance of 18.300km. The nearest neighbor ratio is R = 0.411<1, Z-value is -33.626, and P-value is 0.000, indicating a significant clustered distribution of the spatial structure of TVs in the YRB. To verify the accuracy and reliability of this result, and to more objectively represent the dispersion and clustering of TVs, a kernel density analysis (Fig 2A) was conducted. The spatial distribution of TVs is uneven, showing a clear pattern of "general dispersion with local concentration." Spatial clustering is primarily characterized by grouped distribution, with major clustering centers in Shanxi, Shaanxi, and Qinghai provinces. Three major clustering areas are formed in the central-eastern part of Shanxi Province, the northern and southern parts of Shaanxi, and the southeastern part of Qinghai Province. Four secondary clustering areas are formed in the Ningxia Guyuan region, Inner Mongolia Baotou region, Henan Jiaozuo region, and Shandong Zibo region, along with several sparsely populated clustering areas in other regions. The cold and hot spot clustering analysis (Fig 2B) shows that cold and hot spots largely coincide with kernel density clustering areas. Hot spots are formed in the Shanxi Jincheng—Changzhi—Lüliang region, and the Qinghai Xining-Haidong region. Secondary hot spots are formed in Qinghai Huangnan, Gansu Linxia and Gannan, Shanxi Linfen, and Henan Jiaozuo. Cold spots are formed in the Shandong Jinan—Zibo region, with other secondary cold spots being more dispersed, distributed across nine provinces. Overall, the southern and eastern regions are hot, while the northern and central regions are cold, indicating a greater degree of clustering in the southern and eastern regions compared to the northern and central regions.

The YRB is a concentration area of TVs in northern China, accounting for approximately 10.93% of the total number of TVs in the country. As indicated in Table 2, regionally, the middle reaches of the basin have the highest number and most concentrated distribution of TVs, totaling 547, which constitutes 61.32% of the basin’s TVs. This is followed by the upper and lower reaches, with 262 TVs in the upper reaches, accounting for 29.37% of the total, and 83 in the lower reaches, accounting for 9.30%. Overall, the YRB exhibits a spatial distribution pattern characterized by "more in the east, less in the west; more in the south, less in the north." The TVs are mainly distributed in the middle and upper reaches of the Yellow River, forming two high-density distribution areas in these regions.

**Table 2:** Statistics on the distribution of TVs in the YRB.

Area	Province(autonomous)	TVs / unit	Proportion / %
Upstream	Qinghai	153	17.15
	Sichuan	0	0.00
	Gansu	55	6.17
	Ningxia	24	2.69
	Inner Mongolia	30	3.36
	Subtotal	262	29.37
Onstream	Shaanxi	121	13.57
	Shanxi	426	47.76
	Subtotal	547	61.32
Downstream	Henan	63	7.06
	Shandong	20	2.24
	Subtotal	83	9.30
	Total	892	100.00

#### 4.1.2 Distribution characteristics by category

**Fig 3:**
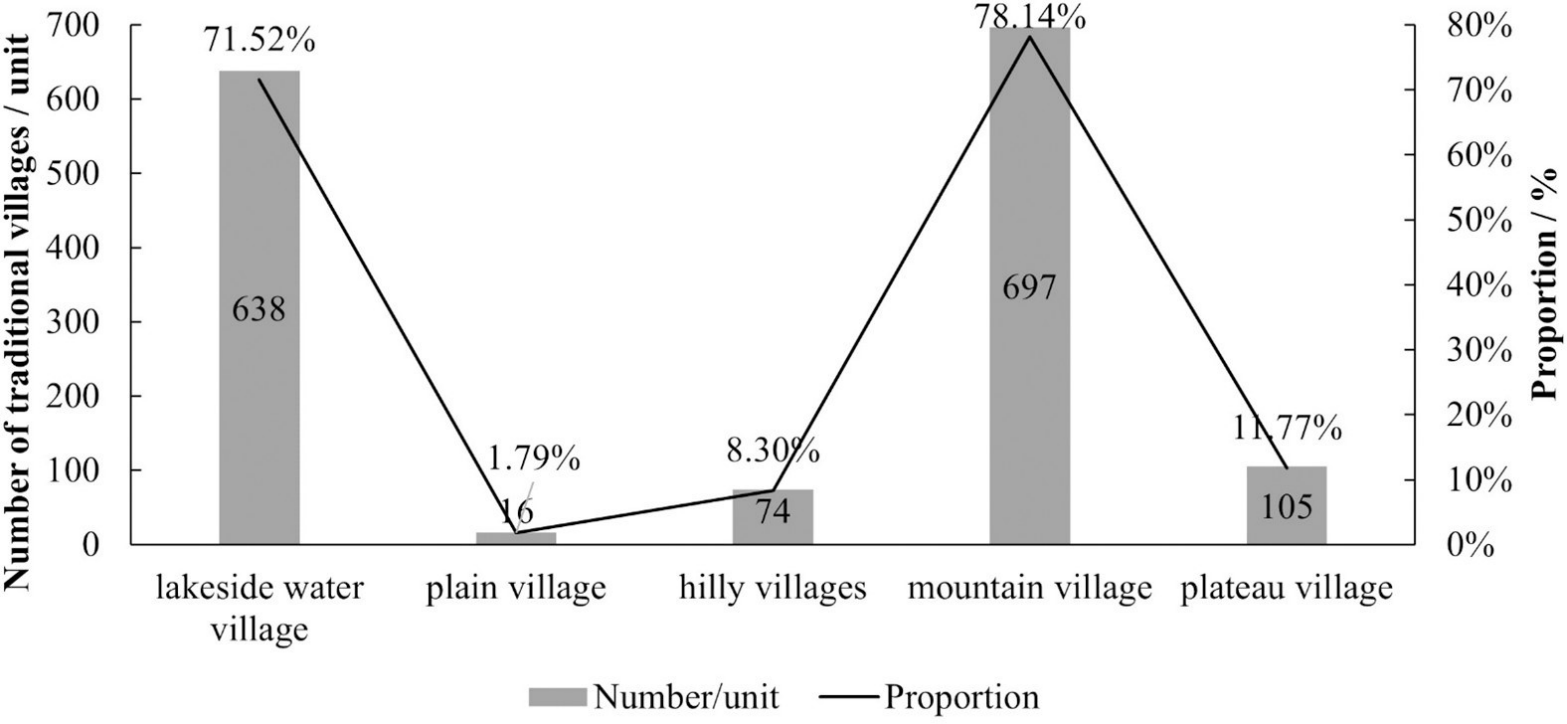
The number of different types of TVs in the YRB.

The formation and evolution of TVs are directly influenced by natural geographical conditions. Different altitudinal regions have different types of village distributions. Therefore, this paper adopts a natural geographical perspective and, drawing from the experience of natural zoning, roughly categorizes TVs into five types: lakeside water village, plain villages, hilly villages, mountain villages, and plateau villages. Looking at the proportion of traditional village types within the basin (Table 3 and Fig 3), there are 638 lakeside water village, accounting for 71.52% of the total TVs in the entire basin. This is due to the numerous tributaries of the Yellow River, the developed water system, and the wide distribution of lakes, resulting in a larger number of TVs in these lakeside water village. Geographically, mountain villages are the most numerous within the YRB, totaling 697, which accounts for 78.14% of the total. Next, there are relatively more plateau villages, with 105 in total, making up 11.77% of the overall count. This is because the terrain of the YRB is highly undulating, predominantly characterized by plateaus and mountains, hence a relatively larger number of TVs are distributed here. In contrast, terrains like plains and hills are less common, leading to a smaller proportion of TVs.

**Table 3:** Type division and quantity statistics of TVs.

Elevation/m	Category	Number/unit	Proportion/%
0–200	Lakeside water village	638	71.52
<200	Plain village	16	1.79
200–500	Hilly villages	74	8.30
500–2500	Mountain village	697	78.14
>2500	Plateau village	105	11.77

### 4.2 Spatio-temporal evolution analysis

**Fig 4:**
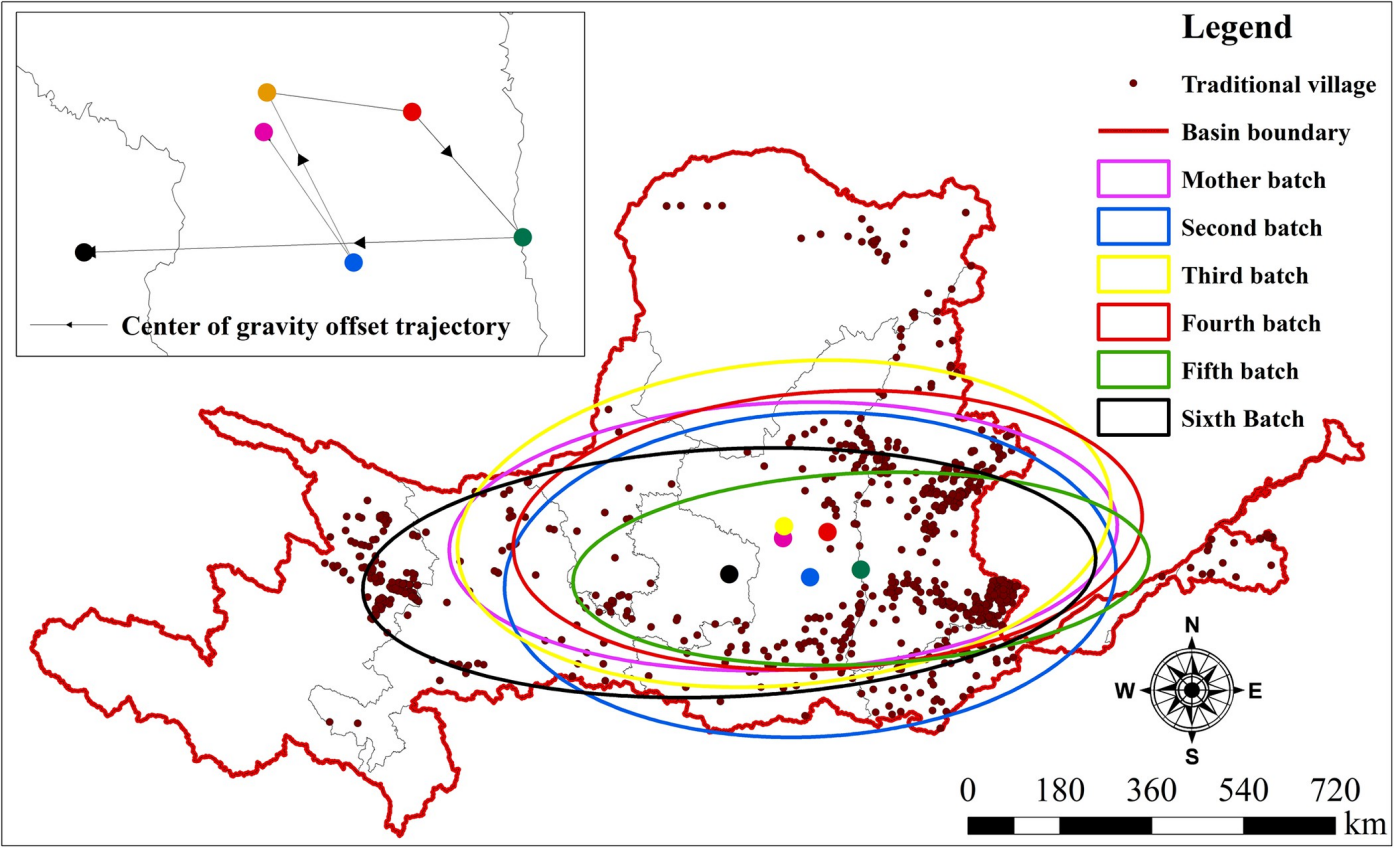
SDE and centroid distribution of TVs in the YRB at different times. The maps were generated by ArcGIS 10.8 and were for illustrative purposes only.

This study employs elliptical standard deviation and centroid change analysis to reveal the spatiotemporal distribution patterns and their evolution in TVs of the YRB. By analyzing the SDE and centroid distribution of TVs in different phases (Fig 4), the results indicate a trajectory of the ellipse centroid moving first from north to south, then back to the north, and finally shifting from east to west. The distribution center of the first batch of TVs was in the Yan’an area of Shaanxi Province, located in the middle-eastern part of the YRB. The second batch showed a significant shift in centroid, moving south to the southern part of Yan’an. Then, in the third batch, it moved northward and eastward in the fourth batch, indicating a clear eastward development trend. The centroid of the fifth batch significantly moved from the north to the south, located at the border between Shaanxi and Shanxi provinces. In the sixth batch, the centroid underwent its largest change, rapidly moving west to Qingyang in the Longdong area of Gansu, suggesting a gradual convergence of the centroid’s trajectory towards the central-western part, with a general shift towards the central area of the YRB. According to the results of Table 4, from an azimuthal perspective, the elliptical distribution direction of TVs from batches 1–6 was uniformly "east-west." As the number of batches increased and time progressed, the major axis generally first shortened and then lengthened, while the minor axis initially lengthened and then shortened. This illustrates that within the basin, TVs initially dispersed and later concentrated, with their spatial distribution gradually becoming more balanced. In summary, the spatial distribution of TVs within the YRB initially concentrated in the northern Shaanxi region. Subsequently, by the fifth batch, they rapidly expanded southward into the Shanxi-Shaanxi region and gradually evolved towards the central and western regions in subsequent batches. The distribution became progressively more balanced, with the centroid gradually shifting towards the central area of the YRB.

**Table 4:** Characteristic ellipse parameters of TVs in different batches of the YRB.

Historical Year	Long Semi-Axis / km	Short Half-Axis / km	Center Coordinates	Azimuth/°
Mother batch	5.885	1.898	(109°7′E,36°30′N)	88.112
Second batch	5.383	2.318	(109°36′E,35°56′N)	88.091
Third batch	5.763	2.290	(109°8′E,36°40′N)	86.257
Fourth batch	5.541	1.966	(109°54′E, 36°35′N)	87.406
Fifth batch	5.073	1.367	(110°29′E,36°3′N)	87.881
Sixth Batch	6.457	1.770	(108°10′E,35°59′N)	87.914

## 5. Spatio-Temporal distribution patterns and evolution analysis

### 5.1 Natural geographical factors

#### 5.1.1 Topography and terrain

**Fig 5:**
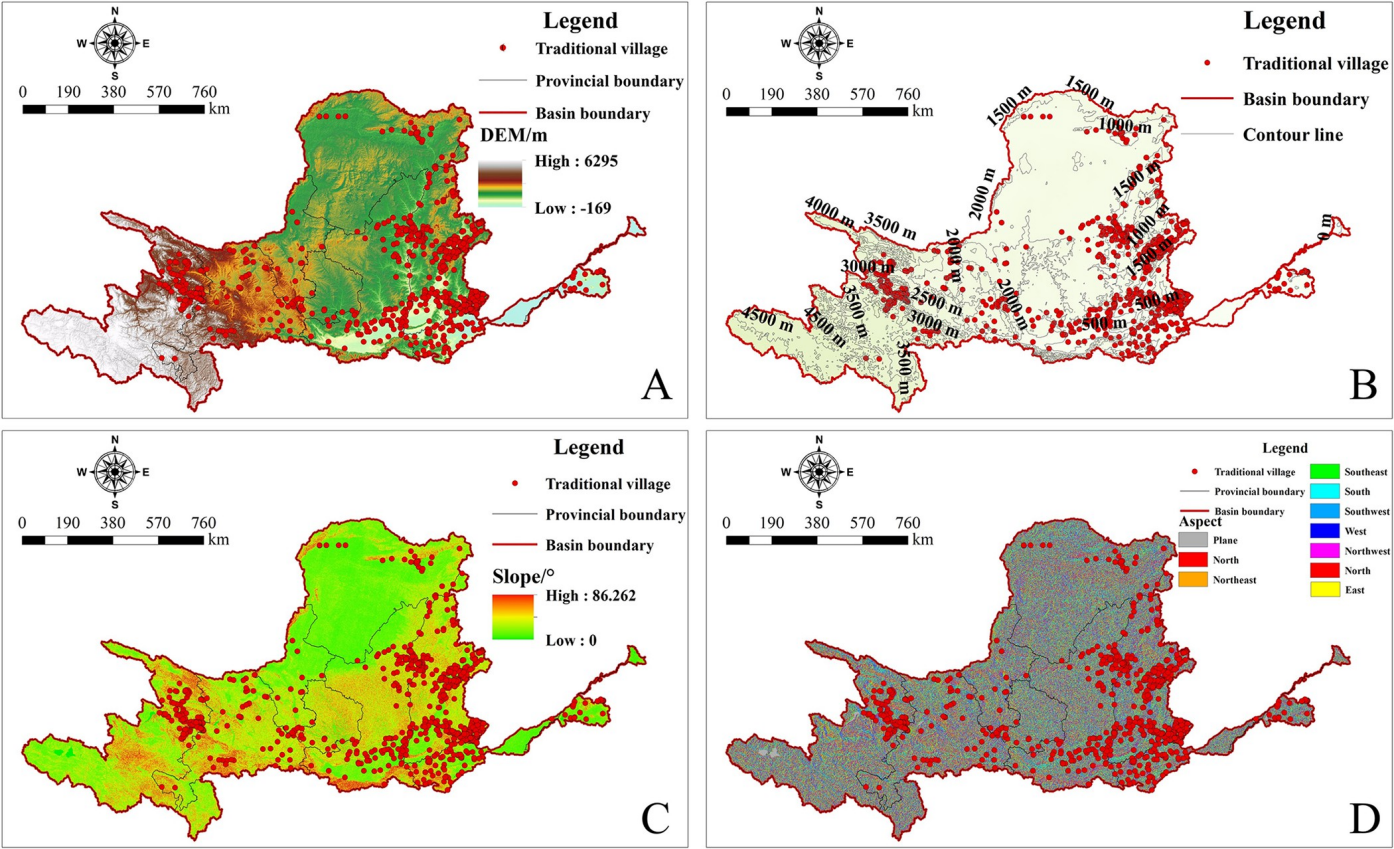
The distribution of elevation, contour, slope and aspect of TVs in the YRB. The maps were generated by ArcGIS 10.8 and were for illustrative purposes only.

The YRB is located on four major geomorphologic units: the Tibetan Plateau, the Loess Plateau, the Inner Mongolia Plateau, and the North China Plain, as well as on three major terraces of Chinese topography. The complex and diverse topography provides foundational support for the formation of TVs. This paper, using ArcGIS, overlays the TVs of the YRB with topography, referring to "The 1:1,000,000 Geomorphological Mapping Standards of China" (Trial) [72], and reclassifies the topography into five types: hills, low mountains, middle mountains, high mountains, and very high mountains, see Table 5. Combining Fig 5A and 5B, it is evident that the lowest elevation in the YRB is -169m, and the highest is 6295m. There are 99 TVs at elevations below 500m, accounting for 11.10% of the total; the most are distributed between elevations of 500-1000m, numbering 416 and accounting for 46.64%; there are 385 villages at elevations of 1000-3500m, representing 43.16%; and only 2 at elevations above 3500-5000m, making up 0.22%. This indicates that the spatial layout of TVs in the YRB is significantly biased towards lower elevations, mainly distributed in low mountain areas.

**Table 5:** Statistics of different altitude distribution of TVs in the YRB.

Range/m	Terrain	Number/unit	Proportion/%
<500	Hill	99	11.10
500–1000	Low mountain	416	46.64
1000–3500	Medium mountain	385	43.16
3500–5000	High mountains	2	0.22
>5000	Higher mountains	0	0.00

By extracting slope data of TVs from DEM (Fig 5C) and classifying them into six categories, combined with Table 6, it is found that the majority of TVs are located in plains or gently sloping areas with slopes below 13°. Among them, there are 463 TVs with slopes ranging from 0 to 6.4°, accounting for 51.91% of the total. There are 319 TVs with slopes ranging from 6.4 to 13.14°, accounting for 35.76% of the total.

**Table 6:** Statistics of different slope distribution of TVs in the YRB.

Range/°	Number/unit	Proportion/%
0–6.402	463	51.91
6.402–13.142	319	35.76
13.142–20.555	84	9.42
20.555–29.990	20	2.24
29.990–86.262	6	0.67

Using DEM to generate slope aspect (Fig 5D) and reclassifying the aspect into ten directions, it is found that most TVs within the basin are located on south-facing or west-facing slopes, totaling 623, nearly 70% of the total,see Table 7. Among these, the majority are on south-facing slopes, with 441 villages accounting for nearly 50%; followed by west-facing slopes, with 182 villages, making up 20.40%.

**Table 7:** Statistics on the distribution of different slope aspects of TVs in the YRB.

Range/°	Orientation	Number/unit	Proportion/%
-1—0.000001	Plane	1	0.11
-0.000001–22.5	North	57	6.39
22.5–67.5	Northeast	83	9.30
67.5–112.5	East	81	9.08
112.5–157.5	Southeast	139	15.58
157.5–202.5	South	168	18.83
202.5–247.5	Southwest	134	15.02
247.5–292.5	West	87	9.75
292.5–337.5	Northwest	95	10.65
337.5–360	North	47	5.27

The distribution of TVs within the basin is correlated with DEM and slope aspect, and is associated with the presence of ancient lakes and marshes [73]. Over time, factors such as lakes and marshes have altered the terrain, significantly influencing the spatial layout and clustering characteristics of villages. In summary, it is precisely due to the characteristics of low elevation, gentle slopes, and sun-facing orientation that the YRB has become a concentration area for TVs. At the same time, the complex and diverse geographical environment of the YRB also provides the fundamental conditions for the survival and construction of TVs.

#### 5.1.2 Ancient pathways of transport

**Fig 6:**
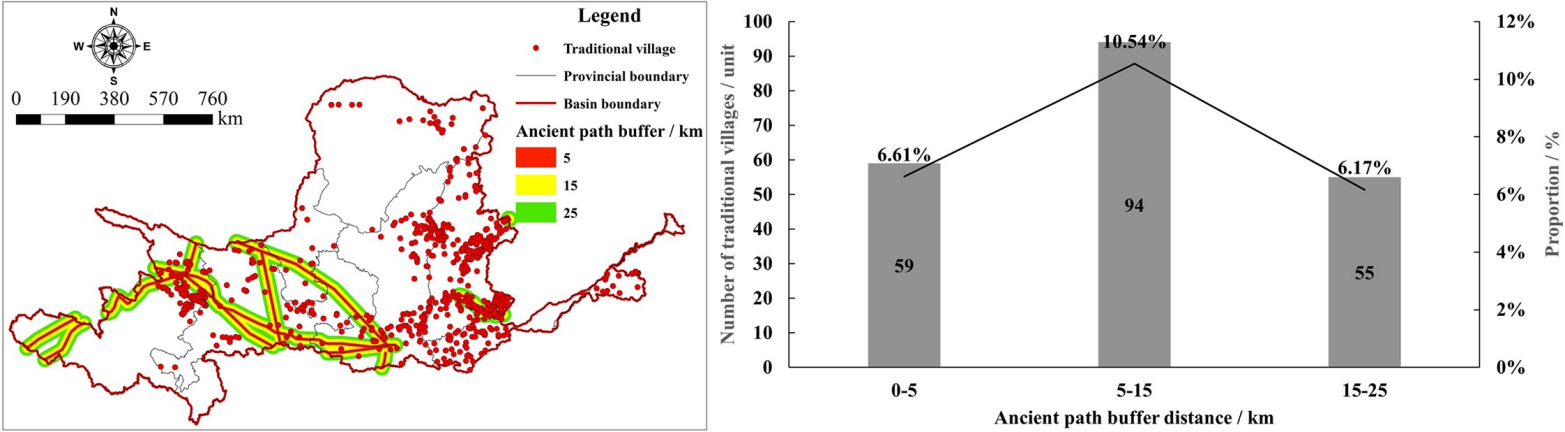
Ancient path buffer analysis of TVs in the YRB. The maps were generated by ArcGIS 10.8 and were for illustrative purposes only.

The history of the ancient Silk Road can be traced back to ancient times and remained active until the 16th century. It mainly connected China by land, passing through Turkey, Iran, and Mesopotamia, and finally reaching the ports along the Mediterranean coast, bringing sustainable cultural tourism to the regions along the route [74]. Therefore, human exchange activities are inseparable from road transportation, and the formation of TVs is closely related to the ancient roads of China. The YRB, rich in historical and cultural heritage, hosts many renowned ancient pathways such as the Tang-Tibet Ancient Road, the Ancient Silk Road, the Taihang Eight Routes, and the Qinling Ancient Road, which to some extent reflect the coupling relationship between TVs and ancient pathways. This paper utilizes GIS to identify and process ancient roads and cultural relics within the region, reconstructs the landscape of ancient roads [75], further reclassifies traditional ancient roads within the basin in raster format, overlays TVs with traditional ancient roads, and conducts buffer zone analysis (Fig 6). The results show that within 25 km of the ancient pathways in the YRB, there are 208 villages, accounting for 23.31% of the total. Most TVs are primarily concentrated within 5–15 km of the ancient pathways, totaling 94 villages, or 10.54% of the total. TVs within 5 km of the ancient pathways are less common, totaling 59, or 6.61% of the total. Since the traditional ancient pathways mainly pass through the middle and upper reaches of the Yellow River, their number of TVs, compared to the total number of TVs in the upper reaches of the Yellow River (262), accounts for 79.39% of the upper reaches’ villages. This indicates that most TVs are located within 25 km of the ancient pathways, reflecting a certain correlation between TVs and ancient transport routes. The overall coupling relationship between TVs and ancient transport routes reflects the influence of the ancient pathways on the formation and distribution of TVs.

#### 5.1.3 River systems

**Fig 7:**
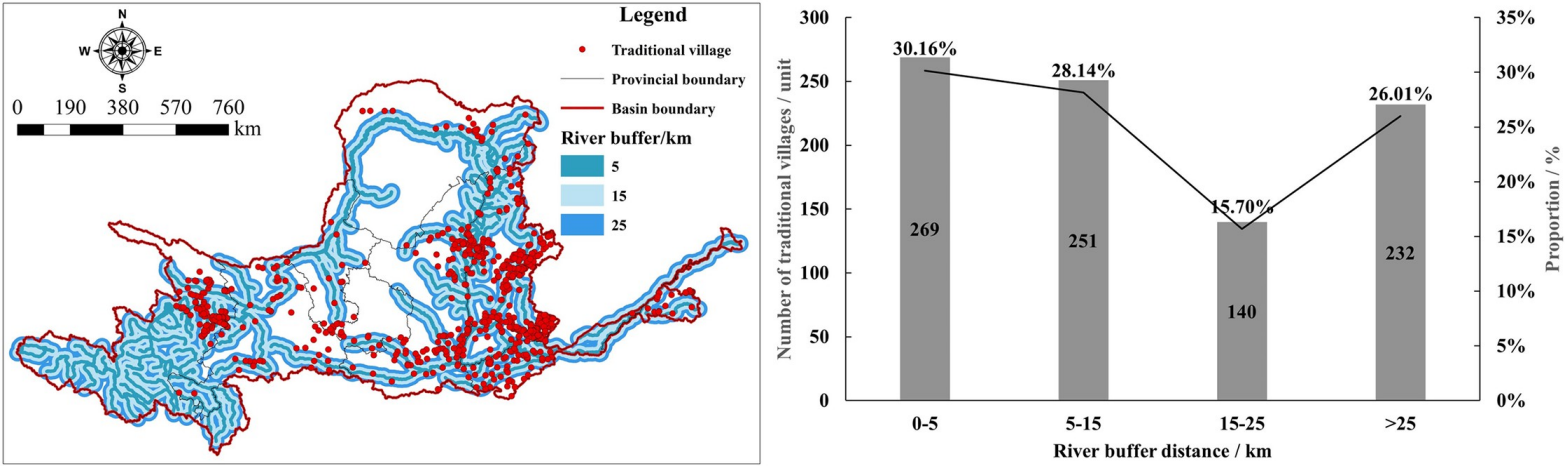
River buffer analysis of TVs in the YRB. The maps were generated by ArcGIS 10.8 and were for illustrative purposes only.

The selection of sites for ancient Chinese villages often followed the principle of "living by the water, choosing water for leisure", integrating the "water element" and exhibiting the characteristic of "gathering water" in traditional settlement culture. A buffer zone analysis of the main rivers and tributaries in the YRB, including the mainstream and its branches (Fig 7), shows that TVs in the YRB are mainly concentrated within 5 km of rivers, with a total of 269 villages, accounting for 30.16% of the total. The next highest concentration is in areas 5–15 km from rivers, with 251 TVs, representing 28.14%. This indicates a clear preference for water proximity in the layout of TVs. However, a negative correlation between the number of TVs and their distance from rivers is also observed in some areas. There are still 232 villages located more than 25 km from rivers, accounting for 26.01% of the total. This suggests that river systems are not the sole determinant factor in the distribution of TVs. Instead, the spatial distribution of TVs is influenced by river systems but is also affected by other factors.

#### 5.1.4 Soil types

**Fig 8:**
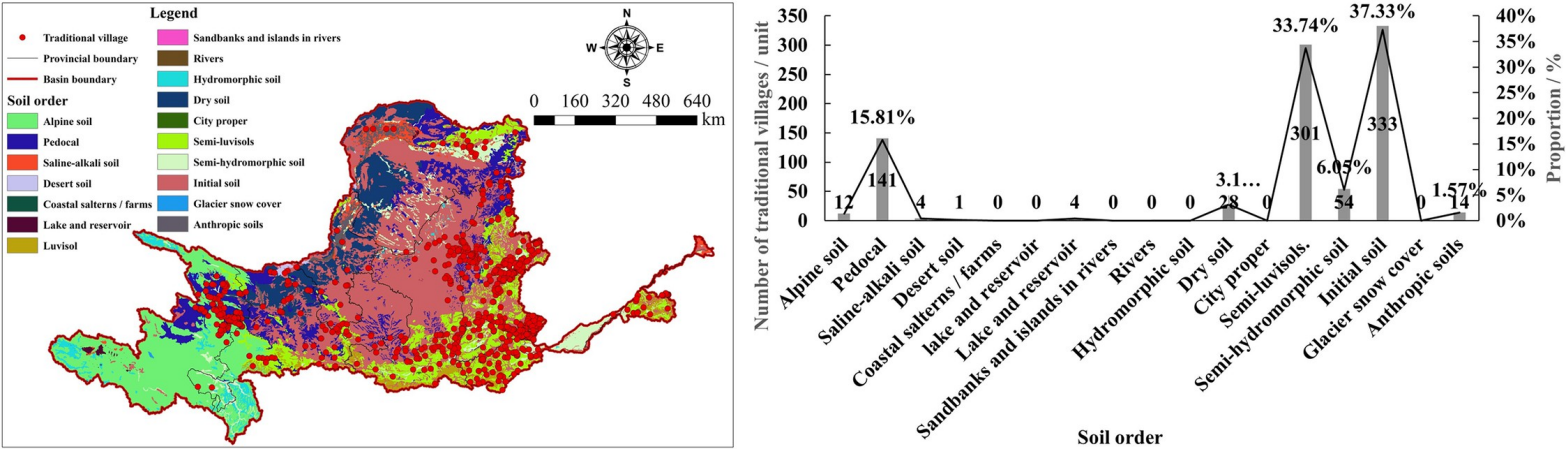
Analysis of soil types of TVs in the YRB. The maps were generated by ArcGIS 10.8 and were for illustrative purposes only.

The YRB, characterized by complex topography and diverse soil types, provides favorable foundational conditions for the growth of TVs. As shown in Fig 8, TVs are predominantly distributed in areas with Initial soils and semi-luvisols, totaling 634 villages, which account for 71.07% of the total. Villages are non-existent in areas covered by glacier snow cover, rivers, coastal salt fields, lakes and reservoirs. Initial soils are mainly found in regions with sparse vegetation and severe soil erosion, while semi-luvisols are primarily located in semi-arid hilly areas. The YRB spans four major geomorphological units and is subject to significant erosion and soil loss due to the perennial activities of the Yellow River, gradually increasing the area of immature and semi-luvisols over time. Additionally, semi-luvisols in the YRB have been developed into farmlands with a long history of cultivation, whereas areas covered by glaciers, snow, rivers, and lakes are unsuitable for the construction of TVs, resulting in fewer village distributions in these areas. It is evident that the vast majority of TVs are located in areas of Initial and semi-luvisols, especially in subcategories such as yellow loess, brown soil, and alluvial soil.

#### 5.1.5 Temperature and precipitation

**Fig 9:**
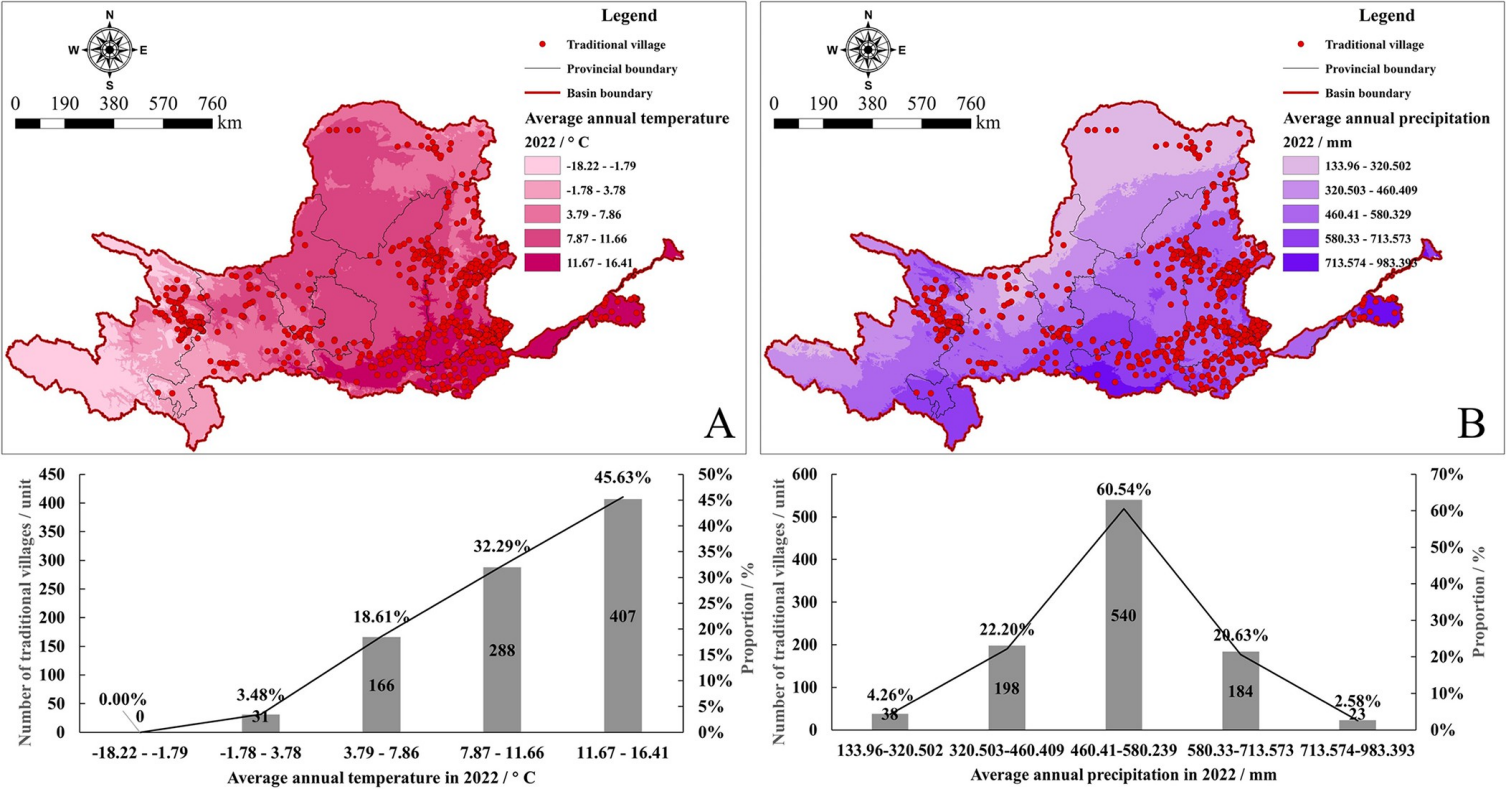
Analysis of temperature and precipitation in TVs in the YRB. The maps were generated by ArcGIS 10.8 and were for illustrative purposes only.

Climate change has a significant impact on human production and living, with favorable climatic conditions and precipitation being crucial for survival and settlement formation [76]. Conversely, if temperature and precipitation conditions fail to meet human survival needs, it will limit the development of architecture, ecology, culture, etc., in the region, thereby affecting the distribution of TVs. Regarding temperature (Fig 9A), the annual average temperature in the YRB ranges between -4 to 14°C, with a general trend of being higher in the south and east, and lower in the north and west. TVs are mainly distributed in areas with temperatures ranging from 11.67 to 16.41°C, accounting for 407 villages or 45.63% of the total. Additionally, TVs are primarily concentrated in regions with mild temperatures, particularly near the lower reaches of the Yellow River, forming a continuous and concentrated distribution pattern. There is a clear positive correlation between TVs and temperature; that is, the higher the temperature, the more the number of TVs.

Regarding precipitation (Fig 9B), the annual average rainfall in the YRB is around 555mm, generally increasing from northwest to southeast. Precipitation significantly decreases in the Inner Mongolia Plateau, while the North China Plain receives more rainfall. The majority of TVs, 540 in total, accounting for 60.54% of all villages, are concentrated in areas receiving 460.41 to 580.239mm of rainfall. TVs are also mainly clustered in regions with abundant rainfall, near the middle and lower reaches of the Yellow River. Overall, temperature and rainfall profoundly influence the spatiotemporal evolution of TVs. Villages in the YRB are highly responsive to climate change, with clusters of TVs densely distributed in areas with relatively warm climates and sufficient rainfall.

### 5.2 Socio-cultural factors

#### 5.2.1 Population and economy

**Fig 10:**
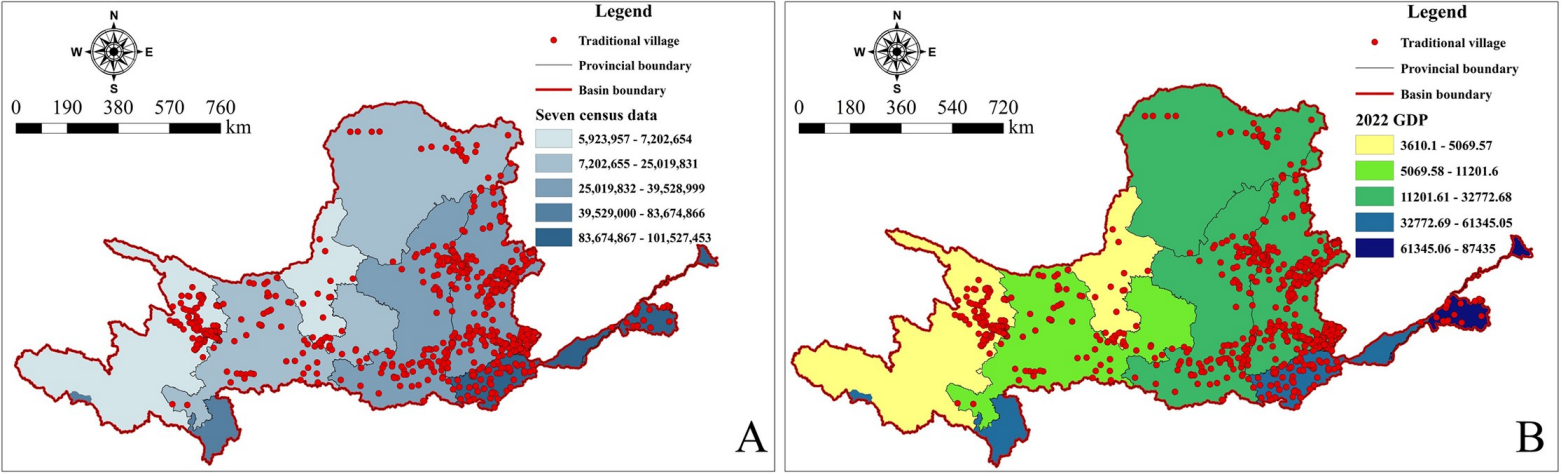
Analysis of population and economic in TVs in the YRB. The maps were generated by ArcGIS 10.8 and were for illustrative purposes only.

Population is a prerequisite for regional economic development. The economy exerts both a siphoning and driving effect on population. Generally, the more concentrated the population, the more developed the economy, and both population and economic factors significantly influence the distribution of TVs. Overlay analysis of population numbers and TVs in the YRB (Fig 10A) shows that the population is mainly concentrated in the middle and lower reaches of the basin, accounting for 630 TVs, or 70.63% of the total. Provinces in these regions have rapidly growing populations and more developed economies. An overlay analysis of economic level and TVs (Fig 10B) reveals that the middle and lower sections of the YRB, including Shaanxi, Shanxi, Henan, and Shandong provinces, had a GDP exceeding 2 trillion yuan in 2022, indicating a dense distribution of TVs in these areas. Combining population and economic factors, the coupling relationship between TVs and population, and between TVs and the economy, are largely aligned. This demonstrates that the number of people and the level of economic development significantly influence the spatiotemporal evolutionary pattern of TVs.

#### 5.2.2 Ethnic culture

The YRB, expansive and spanning nine provinces, is culturally diverse and rich. The basin is home to several ethnic minorities, including the Hui, Tibetan, Mongolian, Salar, Tu, Dongxiang, and Bao’an, accounting for about 10% of the population. These ethnic groups have rooted their existence in the YRB, and ancient villages have gradually formed amidst the blend of diverse ethnic cultures. The unique geographical environment and way of life have endowed the people on both sides of the river with fascinating folk customs, laying a foundation for the cultural connotations of TVs. In terms of cultural attributes, the Yellow River culture includes agricultural culture, grassland culture, Silk Road culture, minority culture, and marine culture. The diversity, openness, and inclusiveness of ethnic cultures have contributed to the varied cultural forms of TVs. Different areas within the basin also have distinct folk cultures. The middle and upper reaches of the Yellow River belong to the Loess Plateau, nurturing the Yellow River culture. The ancient Silk Road mainly passes through these regions, making the formation and distribution of TVs in these areas closely linked to the Yellow River and Silk Road cultures. The lower reaches are mostly alluvial plains, where the unique natural environment fosters pastoral, agricultural, and marine cultures. Therefore, the formation and distribution of TVs in the lower reaches are closely related to agricultural and marine cultures.

#### 5.2.3 River basin civilization

The primary characteristic of the YRB civilization is its agricultural civilization. The millennia-long agricultural culture has made the YRB the most developed region for agricultural culture in northern China. Agricultural culture is the foundation for the development of villages, and historical culture is an important carrier for the inheritance and continuation of traditional village culture. The basin has fostered a paradigm of harmony between nature and humanity and congruence between home and state, nurturing the unique culture and arts of the Yellow River, including Confucianism and Taoism, and leaving behind many cultural heritages. The YRB has a long history of ancient settlements, with maternal clan village sites from primitive societies formed within the basin. These sites provide natural foundational conditions for the aggregation and preservation of TVs. In terms of the spatiotemporal distribution pattern, TVs are the material carriers of river basin civilization, and their spatial layout is inevitably influenced by cultural factors within the basin. Yellow River culture includes not only agricultural culture but also northern pastoral civilization, ritual and music civilization, concepts of space and time, and various philosophical doctrines. These elements are key to the formation and distribution of TVs, making river basin civilization an important factor influencing the spatiotemporal distribution pattern of TVs.

## 6. Discussion

### 6.1 Interaction detection analysis of influencing factors

Factor detection is used to explore the driving forces between two independent variables, identifying the dominant factors affecting the spatial differentiation of TVs. Based on the analysis of driving factors, the spatial distribution of TVs is influenced by a combination of factors. The study considers temperature, soil, population, economy, precipitation, ancient paths, altitude, aspect, rivers, and slope as independent variables (*x*), with the kernel density of TVs as the dependent variable (*y*). Using ArcGIS, these variables are classified into six levels based on natural breakpoints and reclassified for discrete analysis. Using Formula 6, the factor interaction results were calculated using GeoDetector, as shown in Table 8, thus assessing their impact and explanatory power on the spatial differentiation of TVs in the YRB. The results show that the factors are ranked by their q-values as soil (*x*_2_) > temperature (*x*_1_) > altitude (*x*_7_) > economy (*x*_4_) > precipitation (*x*_5_) > population (*x*_3_) > ancient paths (*x*_6_) > rivers (*x*_9_) > slope (*x*_10_) > aspect (*x*_11_). From the p-value analysis, soil, temperature, and altitude have a p-value of 0, indicating their significant influence. Hence, natural geographical conditions such as soil, temperature, and altitude are the primary factors influencing the distribution of TVs in the YRB, followed by economy, precipitation, and population. Aspect has the least impact. Due to the complex terrain and varied landscape of the YRB, where agricultural culture thrives, residents historically have chosen to settle in areas with suitable temperatures, lower altitudes, gentle slopes, and fertile soil, leading to a long history and large number of TVs in these regions. Located in the north of China with higher latitudes, most houses in the YRB face south to the sun, making aspect the least influential factor among the many affecting TVs.

**Table 8:** Geographical exploration results of factors influencing spatial differentiation of TVs in the YRB.

Serial Number	Detection Factor	Index Factors	*q* statistic	*p* value
*x*_1_	Temperature	Average annual temperature/°C	0.015	0.000
*x*_2_	Soil	Soil type	0.028	0.000
*x*_3_	Population	Number of population / ten thousand	0.004	1.000
*x*_4_	Economy	GDP / yuan	0.006	0.209
*x*_5_	Precipitation	Average annual precipitation / mm	0.006	0.027
*x*_6_	Ancient path	Ancient path distance / km	0.004	0.061
*x*_7_	Altitude	Altitude elevation / m	0.011	0.000
*x*_8_	Aspect	Orientation	0.001	1.000
*x*_9_	River	River distance / km	0.003	0.000
*x*_10_	Slope	Degree / °	0.002	0.594

**Note:** The larger the *q* value, the stronger the driving force of the factor, and the smaller the *p* value, the more significant the result.

**Fig 11:**
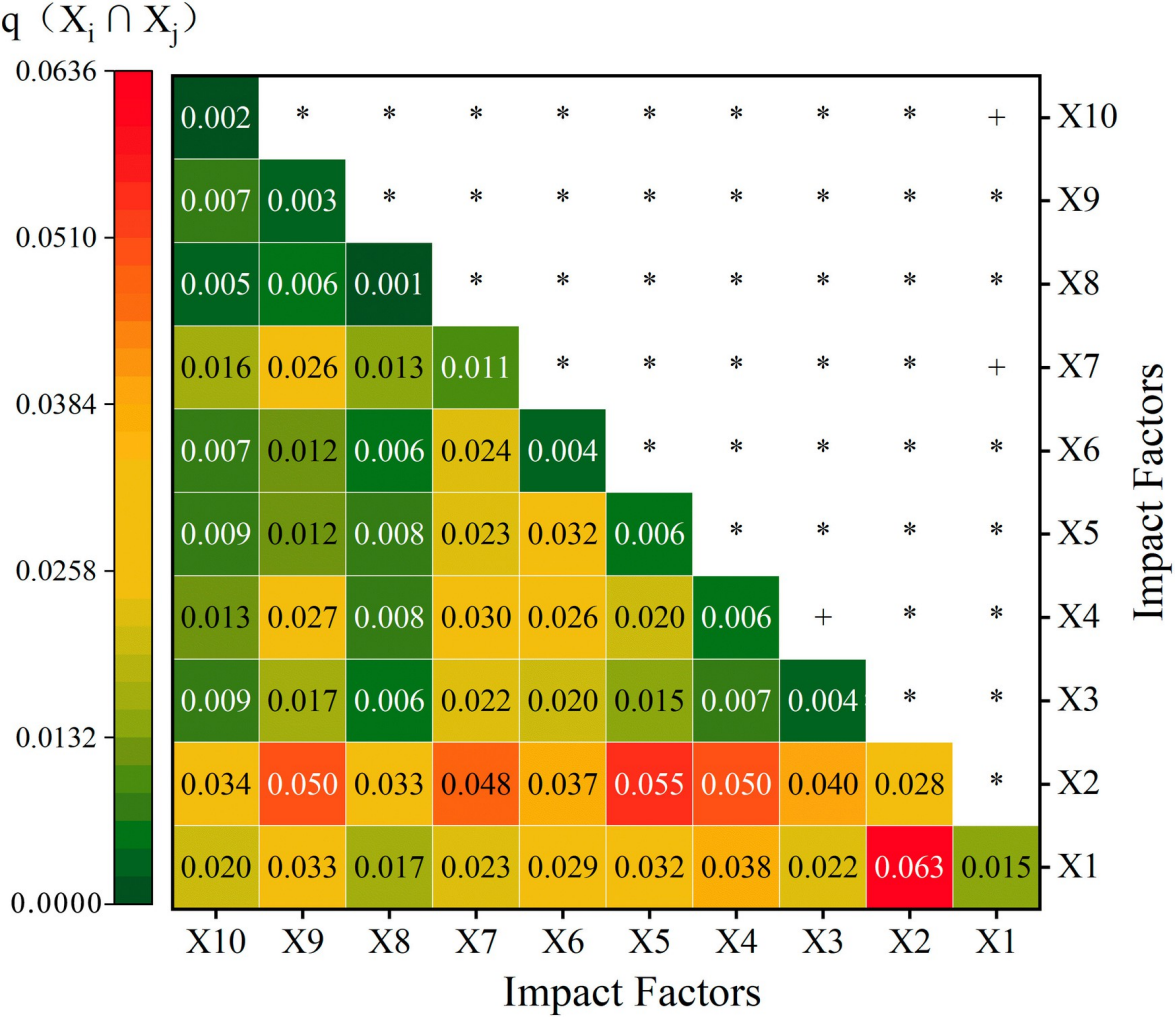
The interaction test results of the influencing factors of the spatial distribution of TVs in the YRB. **Note:** * denotes nonlinear enhancement [*q*(*x*_*i*_∩*x*_*j*_)>(*x*_*i*_+*x*_*j*_)]; + denotes two-factor interaction enhancement [*q*(*x*_*i*_∩*x*_*j*_)>Max (*x*_*i*_,*x*_*j*_)].

Building on the results of the single-factor detection, multi-factor interaction detection was conducted for the spatial distribution of TVs in the YRB as per Table 1, leading to the findings of spatial distribution differentiation interaction detection of TVs in the YRB (Fig 11). The results indicate that the interaction types in the YRB primarily comprise of bifactor enhancement and nonlinear enhancement, with the interaction being predominantly nonlinear. The driving force, explanatory power, and influence of bifactor interactions are more significant than those of single factors. The values of *q*(*x*_2_∩*x*_1_)、*q*(*x*_2_∩*x*_5_)、*q*(*x*_2_∩*x*_4_)、*q*(*x*_2_∩*x*_9_)、*q*(*x*_2_∩*x*_7_) are all greater than 0.050, overall showing a nonlinear enhancement trend, with their influence and explanatory power far exceeding that of single factors. The interaction between soil and temperature is the most significant, with an interaction q-value of 0.063; followed by soil with precipitation, economy, rivers, and altitude, with q-values between 0.05–0.06. This analysis further illustrates that soil is a key factor in the interactions, and natural geographical conditions are the fundamental factors influencing the spatial differentiation of TVs in the YRB, with soil playing a dominant role in these fundamental factors. Ancient site selection often favored areas suitable for agricultural activities, with TVs densely distributed in regions with abundant precipitation, developed economies, proximity to rivers, lower altitudes, and suitable temperatures, which are mostly in the middle reaches of the Yellow River. The upper reaches of the Yellow River, with its arid climate and low precipitation, are not conducive to agricultural production and settlement formation, while the lower reaches with more precipitation are prone to frequent floods, unfavorable for the long-term preservation of TVs.

In conclusion, soil exerts the strongest influence and explanatory power on the spatial distribution of TVs in the YRB, followed by precipitation, economy, rivers, and altitude. Soil becomes the primary factor influencing the spatial distribution of TVs in the YRB due to the region’s fragile ecological environment, complex terrain, extensive gullies, severe soil erosion, exposed land surface, and distinct dry seasons. Throughout history, the local residents in the area have predominantly relied on agriculture for their livelihoods and survival, making the YRB an important agricultural origin. Consequently, these settlements mostly choose areas with fertile loess plateau soil, which are conducive to residential construction. On the other hand, the Yellow River is considered the "Mother River" of China, and generations of people in the basin have been engaged in flood control and irrigation, laying a solid foundation for water management systems and attracting a large population to settle in the area. However, in recent years, the protection efforts in the YRB have not been effectively implemented. Therefore, attention should be focused on the protection of soil within the basin, restoring the capacity of vegetation to conserve water and soil, reducing excessive land reclamation and grazing, promoting high-quality development in the YRB, and thereby enhancing the protection of TVs. Additionally, as shown in Fig 11, except for the dual-factor enhancement of *x*_2_∩*x*_1_,*x*_1_∩*x*_7_, and *x*_1_∩*x*_10_, the rest exhibit non-linear enhancement. Among them, the prominent factors are temperature, soil, population, and economy, with soil occupying the most significant position. This further indicates that soil is the primary factor influencing the spatial distribution of TVs in the YRB.

### 6.2 Driving factors affecting TVs in the river basin

The study reveals that TVs in the YRB exhibit a distinct spatiotemporal distribution pattern characterized by "overall dispersion and local concentration", a result of the combined influence of physical geography and socio-cultural factors. Analysis indicates that natural geographical factors are the primary influence on village distribution, forming the basis and intrinsic conditions for village location, thus fundamentally determining the spatial distribution of villages. This aligns with previous research and field observations. Firstly, the spatial distribution of TVs in the YRB is closely related to soil factors among natural elements. Given the region’s long-standing agricultural culture, diverse soil types, and extensive farming history, soils like loess, brown earth, and alluvial soil have facilitated agricultural production and settlement location, leading to a higher concentration and number of TVs in the basin. Secondly, there are significant differences in the spatial distribution of TVs in the upper, middle, and lower reaches of the YRB. In the upper reaches, the climate is arid with less precipitation, and TVs tend to be located in areas with more suitable temperatures, more rainfall, and closer to rivers, such as the Gan-Qing Hehuang region. The middle reaches, with suitable temperatures and developed economies, have a wider basin area, resulting in concentrated and contiguous distribution of TVs. In contrast, the lower reaches experience more rainfall and frequent flooding, with a smaller basin area, hence fewer TVs. Finally, socio-cultural factors are the external elements influencing the spatial distribution of TVs. They govern the continuity, cultural heritage, and innovation of villages, significantly impacting their development and formation. Basin civilization has laid the foundational conditions for the formation and preservation of traditional village cultures. Nowadays, most applications for TVs within the basin are predominantly culture-based, making the YRB’s culture a key factor in the formation and development of TVs.

The paper analyzes and discusses the distribution characteristics and influencing factors of TVs in the YRB, considering both natural geography and socio-cultural aspects. However, the analysis and study do not address the criteria for traditional village selection, lacking qualitative analysis of architectural structures, clan kinship, regional culture, and local areas. Therefore, future studies should employ a combination of qualitative and quantitative, macro and micro, and comprehensive and localized approaches to elucidate the mechanisms behind the spatial distribution of TVs in the YRB.

### 6.3 Verification and limitations of the study

This study aims to verify through analysis:

1. The overall spatiotemporal distribution characteristics and evolutionary trends of TVs in the YRB.
2. Primarily analyze the main and secondary factors affecting the spatial distribution of TVs in the YRB, determine the coupling relationships and correlations between these factors and the distribution trends of TVs, and identify the key influencing factors and their degrees of influence. Estimate which factors have more significant impact, stronger effects, and dominant positions, and summarize and explore the reasons these factors affect the spatial distribution patterns of TVs.
3. Through the interaction of influencing factors, investigate the significant factors affecting the spatiotemporal distribution of TVs in the YRB, detect the relationships between TVs and other elements within the basin, and analyze the enhancing and diminishing effects of pairwise factor interactions on the distribution trends of TVs.
4. The SDE analysis reveals that the azimuth *θ* of TVs in batches 1–6 within the YRB is around 86–88°, showing a general east-west distribution pattern. The lateral distribution trend is evident, with the major axis distances concentrating between 5–6.5 km and minor axis distances between 1–2.5 km. The low ellipticity indicates a "flattened" state, revealing a relatively concentrated and directionally balanced spatial distribution of TVs in the YRB, with minimal changes in spatial evolution trends. The NNI analysis indicates an average observed distance of 7.530 km, with an expected average distance of 18.300 km. The nearest neighbor ratio is *R* = 0.411<1, with *Z* value of -33.626 and a *p* value of 0.000, indicating that TVs in the YRB are relatively close to each other, with an overall concentrated distribution trend. The KDE analysis shows that the spatial distribution of TVs, using TVs as entity points and the YRB boundary as the search radius, is uneven, displaying a distinct "overall dispersion, local concentration" characteristic. Spatial clustering is mainly in the form of clustered distribution, with a maximum *f*(*x*) density value of 171.38, primarily concentrated in the provinces of Shanxi, Shaanxi, and Qinghai, indicating higher density of traditional village distribution. Conversely, as it moves closer to the northern border, the kernel density value *f*(*x*) decreases, indicating lower density of traditional village distribution. The hot and cold spot analysis reveals that TVs in the Jinzhong-Changzhi-Lüliang region of Shanxi and the Xining-Haidong region of Qinghai have $G_{i}^{*}$ values greater than 0, forming clustered hotspots. Conversely, in the Jinan-Zibo area of Shandong, the $G_{i}^{*}$ values are less than 0, forming focused cold spots, with a relatively dispersed distribution.

While analyzing and verifying the spatiotemporal distribution of TVs in the YRB, corresponding limitations and difficulties were also encountered. This paper’s analysis of the spatial distribution characteristics of TVs in the YRB and their influencing factors aims to promote high-quality development in the basin and the inheritance and innovation of cultural resources. However, due to the extensive area and dynamic changes of the YRB, the chosen driving factors lack sufficient temporal testing. With the progress of urbanization and the modernization of agriculture and rural areas, ecological and environmental factors continue to influence the spatial distribution of TVs. This paper, grounded in a holistic perspective, analyzes these factors from a macroscopic viewpoint. However, the analysis of influencing factors on TVs in specific geomorphological units, remote rural areas, and ecological conservation areas is inadequate, lacking practical and specific recommendations. Additionally, analyzing the results of interaction detection in TVs, it is noted that the geographical detection model has a limit on the amount of data it can process. Therefore, the conclusion that soil is a leading factor influencing the spatial distribution of TVs still requires practical validation.

## 7. Conclusions

Utilizing GIS, this study investigates the spatiotemporal distribution characteristics of TVs in the YRB and explores their driving factors, leading to the following conclusions:

1. In terms of spatial distribution, the nearest neighbor index *R*< 1 indicates that the TVs in the YRB are generally clustered, exhibiting a concentrated and contiguous distribution pattern of "more in the east and less in the west, more in the south and less in the north", with dense areas primarily located in the upstream regions dominated by Qinghai Province and the midstream areas along the Shanxi-Shaanxi coast.
2. In terms of temporal distribution, the number and scale of villages generally show an increasing trend. The more recent the year, the more balanced the number and proportion of TVs, with uneven inter-provincial distribution and a higher number of mountain and plateau villages within the basin.
3. In terms of spatiotemporal evolution, the focal point of the traditional village layout in the YRB shifts with each phase, showing a migration pattern from north to south, then back north, and finally from east to west.
4. In terms of influencing factors, physical geographic factors are the primary influence on the spatial distribution of TVs in the YRB, followed by socio-cultural factors. In terms of natural environment, the most significant influences under interactive effects are soil, temperature, and elevation. TVs are predominantly distributed in areas with loess, brown soil, and alluvial soils at elevations around 1000 meters, within 15 km of ancient paths and rivers, with temperatures between 11.67–16.41°C and precipitation ranging from 460.41–580.239 mm. In terms of the human environment, TVs are often located in areas with larger populations, developed economies, and rich cultural heritage.
